# Mild COVID-19 imprints a long-term inflammatory eicosanoid- and chemokine memory in monocyte-derived macrophages

**DOI:** 10.1038/s41385-021-00482-8

**Published:** 2022-03-15

**Authors:** Sina Bohnacker, Franziska Hartung, Fiona Henkel, Alessandro Quaranta, Johan Kolmert, Alina Priller, Minhaz Ud-Dean, Johanna Giglberger, Luisa M. Kugler, Lisa Pechtold, Sarah Yazici, Antonie Lechner, Johanna Erber, Ulrike Protzer, Paul Lingor, Percy Knolle, Adam M. Chaker, Carsten B. Schmidt-Weber, Craig E. Wheelock, Julia Esser-von Bieren

**Affiliations:** 1 Center of Allergy and Environment (ZAUM), Technical University of Munich and Helmholtz Center Munich, 80802 Munich, Germany; 2 Division of Physiological Chemistry 2, Department of Medical Biochemistry and Biophysics, Karolinska Institute, Stockholm, Sweden; 3 The Institute of Environmental Medicine, Karolinska Institute, Stockholm, Sweden; 4 Institute of Molecular Immunology and Experimental Oncology, University Hospital rechts der Isar, Technical University of Munich (TUM), School of Medicine, 81675 Munich, Germany; 5 Institute of Computational Biology, Helmholtz Center Munich, 85764 Neuherberg, Germany; 6 Department of Otorhinolaryngology and Head and Neck Surgery, University Hospital rechts der Isar, Technical University of Munich (TUM), School of Medicine, 81675 Munich, Germany; 7 Department of Internal Medicine II, University Hospital rechts der Isar, Technical University of Munich (TUM), School of Medicine, 81675 Munich, Germany; 8 Institute of Virology, Technical University of Munich (TUM), School of Medicine and Helmholtz Zentrum München, 81675 Munich, Germany; 9 German Center for Infection Research (DZIF), Munich partner site, Munich, Germany; 10 Department of Neurology, University Hospital rechts der Isar, Technical University Munich (TUM), School of Medicine, 81675 Munich, Germany; 11 German Center of Lung Research (DZL), Munich partner site, Munich, Germany; 12 Department of Respiratory Medicine and Allergy, Karolinska University Hospital, 141-86 Stockholm, Sweden; 13 Gunma Initiative for Advanced Research (GIAR), Gunma University, Maebashi, Japan

## Abstract

Monocyte-derived macrophages (MDM) drive the inflammatory response to severe acute respiratory syndrome coronavirus 2 (SARS-CoV-2) and they are a major source of eicosanoids in airway inflammation. Here we report that MDM from SARS-CoV-2-infected individuals with mild disease show an inflammatory transcriptional and metabolic imprint that lasts for at least 5 months after SARS-CoV-2 infection. MDM from convalescent SARS-CoV-2-infected individuals showed a downregulation of pro-resolving factors and an increased production of pro-inflammatory eicosanoids, particularly 5-lipoxygenase-derived leukotrienes. Leukotriene synthesis was further enhanced by glucocorticoids and remained elevated at 3–5 months, but had returned to baseline at 12 months post SARS-CoV-2 infection. Stimulation with SARS-CoV-2 spike protein or LPS triggered exaggerated prostanoid-, type I IFN-, and chemokine responses in post COVID-19 MDM. Thus, SARS-CoV-2 infection leaves an inflammatory imprint in the monocyte/ macrophage compartment that drives aberrant macrophage effector functions and eicosanoid metabolism, resulting in long-term immune aberrations in patients recovering from mild COVID-19.

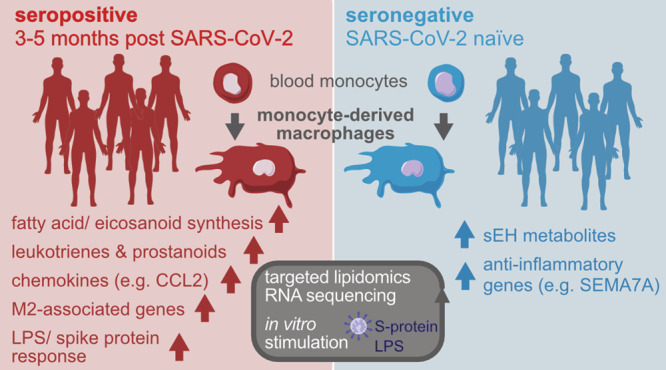

## Introduction

The Coronavirus disease 2019 (COVID-19) has emerged as a global pandemic caused by severe acute respiratory syndrome coronavirus 2 (SARS-CoV-2) infections^1^. Long-term symptoms of COVID-19 are common after severe disease^2^, but may also affect 15–20% of individuals with previous mild disease^3^. Monocyte-derived macrophages (MDM) drive the inflammatory response to SARS-CoV-2 and contribute to cytokine storms in severe COVID-19^4,5^. Severe COVID-19 is associated with profound changes in the myeloid compartment, including expansion of dysfunctional, pro-inflammatory monocytes during the first weeks after SARS-CoV-2 infection^6,7^.

Eicosanoids are bioactive metabolites of polyunsaturated fatty acids (PUFAs) with key roles in infection and inflammation^8^. Eicosanoids are formed from arachidonic acid (AA) through different enzymatic pathways, including the cyclooxygenase (COX) pathway, synthesizing prostanoids and the 5-lipoxygenase (5-LOX) pathway, generating leukotrienes (LTs)^8^. LTs are potent granulocyte-chemotactic metabolites which cause bronchoconstriction, vascular leakage, and airway remodeling^9^. Resident and recruited macrophages in the lung produce high levels of cysteinyl LTs (cysLTs) and leukotriene B_4_ (LTB_4_), thereby promoting granulocyte infiltration, airway inflammation and tissue remodeling^8^. Serum and airway prostanoid- and LT levels are increased in severe COVID-19^10,11^, suggesting a role for eicosanoids in the immune response to SARS-CoV-2 infection.

By studying transcriptome- and lipid mediator profiles in MDM of convalescent SARS-CoV-2-infected individuals with previous mild disease, we show that inflammatory gene expression and eicosanoid profiles as well as altered responsiveness to inflammatory cues are maintained at 3–5 months post infection as well as throughout macrophage differentiation. Pro-inflammatory 5-LOX metabolites were selectively increased in post COVID-19 MDM, suggesting that SARS-CoV-2 infection drives a pro-inflammatory eicosanoid reprogramming that contributes to long-term alterations in innate immune cell function.

## Results and discussion

Recent studies have identified immunological changes in individuals recovering from severe or moderate acute COVID-19 for up to 12 weeks post infection^6,7,12,13^; however potential immune aberrations in the majority of SARS-CoV-2-infected patients, affected by mild disease, have remained obscure.

### Monocyte-derived macrophages of convalescent COVID-19 patients show pro-inflammatory transcriptional reprogramming and enhanced LPS responses

**Fig. 1:**
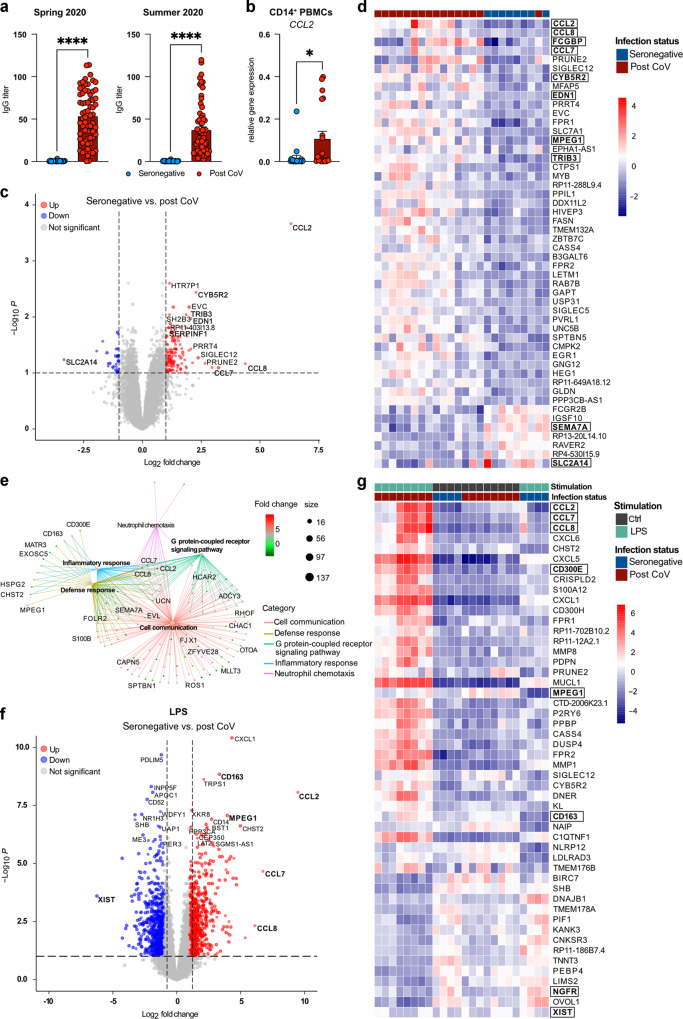
Pro-inflammatory transcriptional reprogramming and heightened LPS response in post COVID-19 MDM. **a** Serum IgG titers of seronegative (*n* = 36) or SARS-CoV-2 seropositive (post CoV) (*n* = 68) individuals in Spring 2020 or at 3–5 months post infection (p.i.) (Summer 2020). Data are shown as mean + SEM. **b** Expression of *CCL2* in CD14^+^ PBMCs of seronegative (*n* = 20) vs. post CoV (*n* = 19) subjects at 3–5 months p.i. **c** Volcano plot showing DEGs between seronegative (*n* = 8) and post CoV MDM (*n* = 16). Top 10 DEGs (base mean > 50), log2 FC > 2 or adjusted *p* value (padj < 0.016 labeled), DEGs with log2 FC > 1 and padj < 0.1 marked. **d** Heatmap of top 50 DEGs between seronegative (*n* = 8) and post CoV (*n* = 16) MDM, padj < 0.1, log2 FC > 1; base mean > 50. **e** GSEA between post CoV (*n* = 8) and seronegative (*n* = 4) MDM + LPS, log2 FC > 2, *p* value < 0.01. **f** Volcano plot showing DEGs between seronegative (*n* = 4) and post CoV (*n* = 8) MDM + LPS. DEGs with log2 FC > 3 or padj < 1 × 10^−6^ labeled, DEGs with log2 FC > 1 and padj < 0.1 marked. **g** Heatmap of top 50 DEGs between seronegative (*n* = 4) and post CoV (*n* = 8) MDM ± LPS, padj < 0.1, log2 FC > 1, base mean > 50. Statistical significance was determined by Mann–Whitney test (**a**, **b**) or DESeq2 (**c**–**f**). *p < 0.05; **p < 0.01; ***p < 0.001; ****p < 0.0001.

Our recent work had shown that patients suffering from chronic airway inflammation exhibit transcriptional reprogramming of MDM^14^, a cell type implicated in COVID-19 pathogenesis^7^. To investigate whether SARS-CoV-2 infection induces persistent changes in MDM gene expression, we studied a sub-cohort from a large SARS-CoV-2 seroprevalence study in healthcare workers^15^ (Table S1, Figs. 1a, S1a). To mimic the pulmonary cytokine milieu, in which infiltrating monocytes differentiate into macrophages, MDM were differentiated in the presence of GM-CSF and TGF-β1^16,17^, which resulted in a similar MDM population in seronegative and seropositive subjects (Fig. S1b).

At 3–5 months after SARS-CoV-2 infection, antibody levels in the seropositive group had dropped by ~30% and 16.2% (vs. 2.8% in the seronegative group) reported persistent symptoms (Figs. 1a, S1c, Table S1). Differential blood cell counts were similar between seronegative and seropositive individuals (Table S1).

*CCL2*, which is increased in monocytes during severe, acute disease^6^, was upregulated in post COVID-19 monocytes, suggesting a persistent inflammatory imprint despite mild disease in the investigated cohort (Fig. 1b).

RNA-sequencing (RNAseq) analysis identified 163 differentially expressed genes (DEGs) in MDM differentiated from monocytes of seropositive individuals 3–5 months post infection compared to MDM from seronegative subjects (Fig. 1c, d, Table S1). Post COVID-19 MDM showed higher expression of pro-inflammatory chemokines (*CCL2*, *CCL8*, *CCL7*), driving neutrophil recruitment, including in COVID-19^18,19^ (Fig. 1c, d, Table S1).

*FCGBP* and endothelin-1 (*EDN1*), implicated in anti-viral defense and pro-fibrotic macrophage activation^20,21^ were also upregulated in post COVID-19 MDM, together with cytochrome B5 reductase 2 (*CYB5R2*), involved in respiratory burst and fatty acid metabolism^22^ (Fig. 1d). In contrast, Semaphorin-7A (*SEMA7A*), implicated in the synthesis of pro-resolving lipid mediators^23^, was downregulated in post COVID-19 MDM (Fig. 1d). Post COVID-19 MDM further showed enhanced inflammatory responses to lipopolysaccharide (LPS), characterized by an exaggerated induction of chemokines involved in neutrophil recruitment^24,25^ (Table S1, Fig. 1e–g). Increased expression of perforin-2 (*MPEG1*) in post COVID-19 MDM at baseline or upon LPS stimulation (Fig. 1d–g) further suggested persistently enhanced interferon (IFN) signaling following SARS-CoV-2 infection^26^. In contrast, expression of nerve growth factor receptor (*NGFR*), X inactive specific transcript (*XIST*) and *SEMA7A*, mediating anti-inflammatory or pro-resolving effects on macrophages^23,27,28^, was reduced in LPS-stimulated post COVID-19 MDM (Figs. 1f, g, S1h, Table S1). Thus, despite mild acute disease in the investigated cohort, MDM exhibited a persistent inflammatory imprint, which was associated with increased symptom burdens and aberrant LPS responses at 3-5 months post infection (Figs. S1c, 1e–g).

### SARS-CoV-2 S-protein-triggered IFN response is exaggerated in post COVID-19 MDM

To define consequences of SARS-CoV-2-induced macrophage reprogramming for re-infection or vaccination, we investigated the response of post COVID-19 MDM to SARS-CoV-2 spike (S)-protein. Entry of SARS-CoV-2 is mainly mediated via recognition of its transmembrane S-glycoprotein by angiotensin-converting enzyme 2 (ACE2) and processing by TMPRSS2^29^. However, *ACE2* and *TMPRSS2* expression in MDM was 100 times lower compared to airway epithelial cells, the major cellular targets of SARS-CoV-2, regardless of inflammatory stimulation or glucocorticoid treatment (Fig. S1d–f). Yet, macrophages can respond to S-proteins of SARS-CoV-1 or SARS-CoV-2 via innate sensing mechanisms including C-type lectins^30,31^, which were upregulated in post COVID-19 MDM (Fig. S1g).

**Fig. 2:**
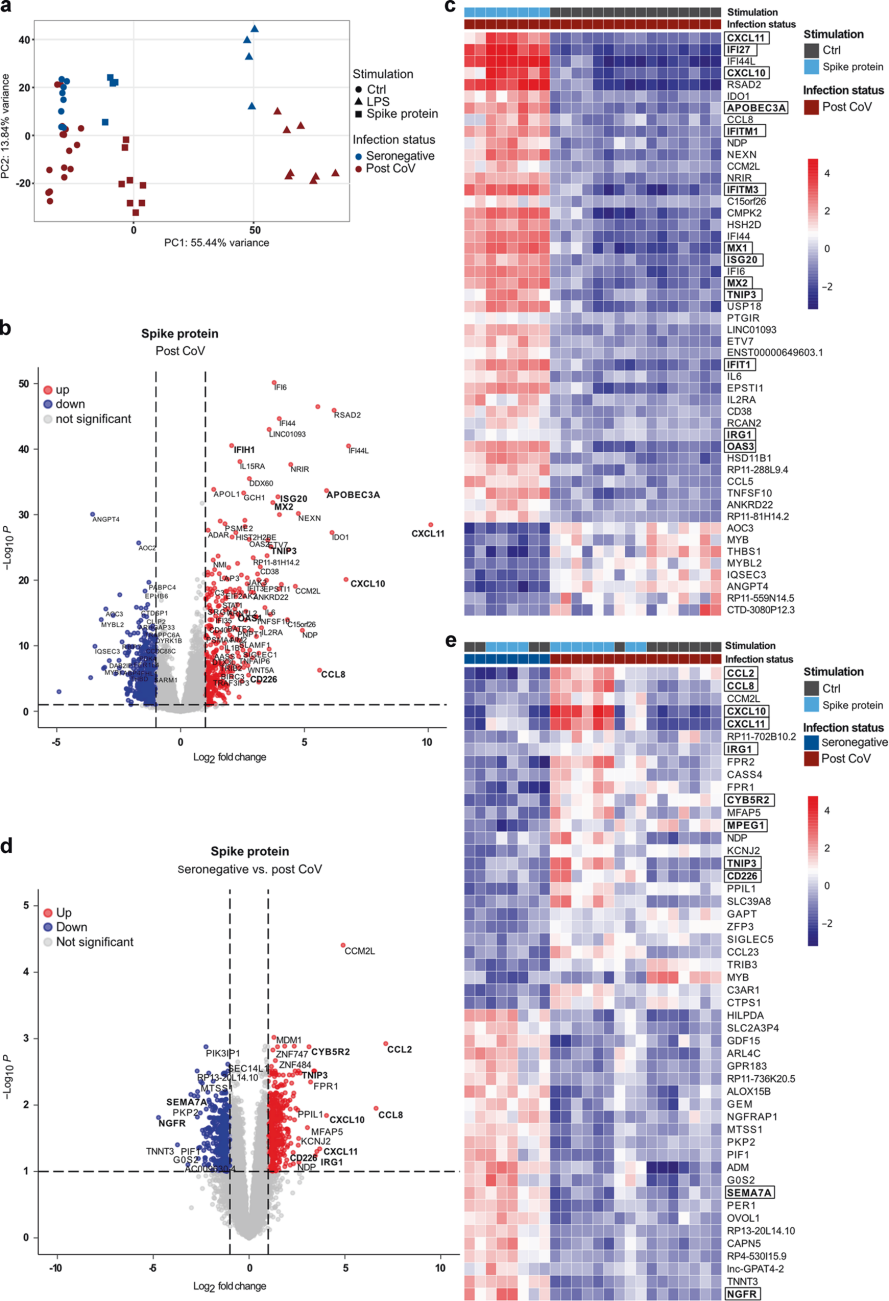
S-protein-induced type I IFN and chemokine responses are exaggerated in post COVID-19 MDM. **a** PCA of RNAseq datasets (baseline, S-protein, LPS) for seronegative (*n* = 4–8) or post CoV (*n* = 8–16) MDM. **b** Volcano plot of DEGs for post CoV MDM (*n* = 8) ± S-protein. DEGs with log2 FC > 5 or padj < 0.00001 (DESeq2) are labeled, DEGs with log2 FC > 1 and padj < 0.1 are colored. **c** Heatmap of top 50 DEGs in post CoV MDM (*n* = 8–16) ± S-protein, padj < 0.1, log2 FC > 1, base mean > 50. **d** Volcano plot of DEGs of S-protein-stimulated MDM from seronegative (*n* = 4) vs. post-CoV (*n* = 8) donors. DEGs with log2 FC > 2.5 or padj < 0.003 are labeled, DEGs with log2 FC > 1 and padj < 0.1 are colored. **e** Heatmap of top 50 DEGs of MDM ± S-protein from seronegative (*n* = 4) or seropositive (*n* = 8) donors, padj<0.1, log2 FC > 1, base mean > 50.

Indeed, MDM readily responded to S-protein and transcriptional differences between seronegative and post COVID-19 MDM were exacerbated by both S-protein and LPS (Fig. 2a). S-protein induced multiple interferon-stimulated genes (ISGs) (e.g. *IFI27*, *IFITIM1/3*, *APOBEC3A, ISG20, MX1/2, OAS1/3*) (Fig. 2b, c, Table S1), demonstrating that it induces an antiviral state in MDM. S-protein stimulation of post COVID-19 MDM resulted in a higher number of DEGs compared to seronegative MDM (858 vs. 220), indicative of a persistently enhanced responsiveness to SARS-CoV-2 several months post infection (Table S1).

The induction of IFN-induced genes (e.g. *CXCL10*, *CXCL11*, *MPEG1*) was increased in S-protein-stimulated post COVID-19 MDM (Fig. 2d, e, Table S1), supporting a role for type I IFN signaling in macrophage reprogramming by SARS-CoV-2 infection. MDM from convalescent SARS-CoV-2-infected subjects showed an enhanced LPS- and S-protein-triggered induction of chemokines (*CCL2*, *CCL8*, *CXCL10*, *CXCL11*) and M2-associated genes (*CD226*, C*D163, CD209*, *TIMP3*, *MERTK*, *TNIP3*), suggesting a pro-inflammatory, T-cell suppressive^32,33^ MDM phenotype (Figs. 1e–g, 2d, e, Table S1). This was in agreement with the exaggerated S-protein- or LPS-mediated induction of immune regulatory enzymes and receptors, including *ACOD1/ IRG1*, *PTGES* and *CD300E* in post COVID-19 MDM (Figs. 1e–g, 2d, e, Table S1)^32,34,35^.

Thus, previous SARS-CoV-2 infection imprints a pro-inflammatory macrophage phenotype, that mounts exaggerated chemokine- and IFN responses, but likely exhibits impaired T-cell stimulatory and pro-resolving capacities. This was in line with previous studies identifying a dysfunctional, pro-inflammatory monocyte activation for up to 12 weeks after SARS-CoV-2 infection^7,13^ and additionally suggested the long-term persistence of a pro-inflammatory macrophage state following mild disease. Changes in gene expression of post COVID-19 MDM were amplified by inflammatory stimuli, suggesting a “trained” state that lasted for at least 5 months post infection. Mechanistically, this may be driven by IFN-mediated reprogramming as post COVID-19 MDM exhibited an exaggerated upregulation of multiple ISGs, including perforin-2 (*MPEG1*), a driver of type I IFN signaling^26^.

### Post COVID-19 MDM produce increased amounts of inflammatory 5-lipoxygenase metabolites at 3–5 months post SARS-CoV-2 infection

**Fig. 3:**
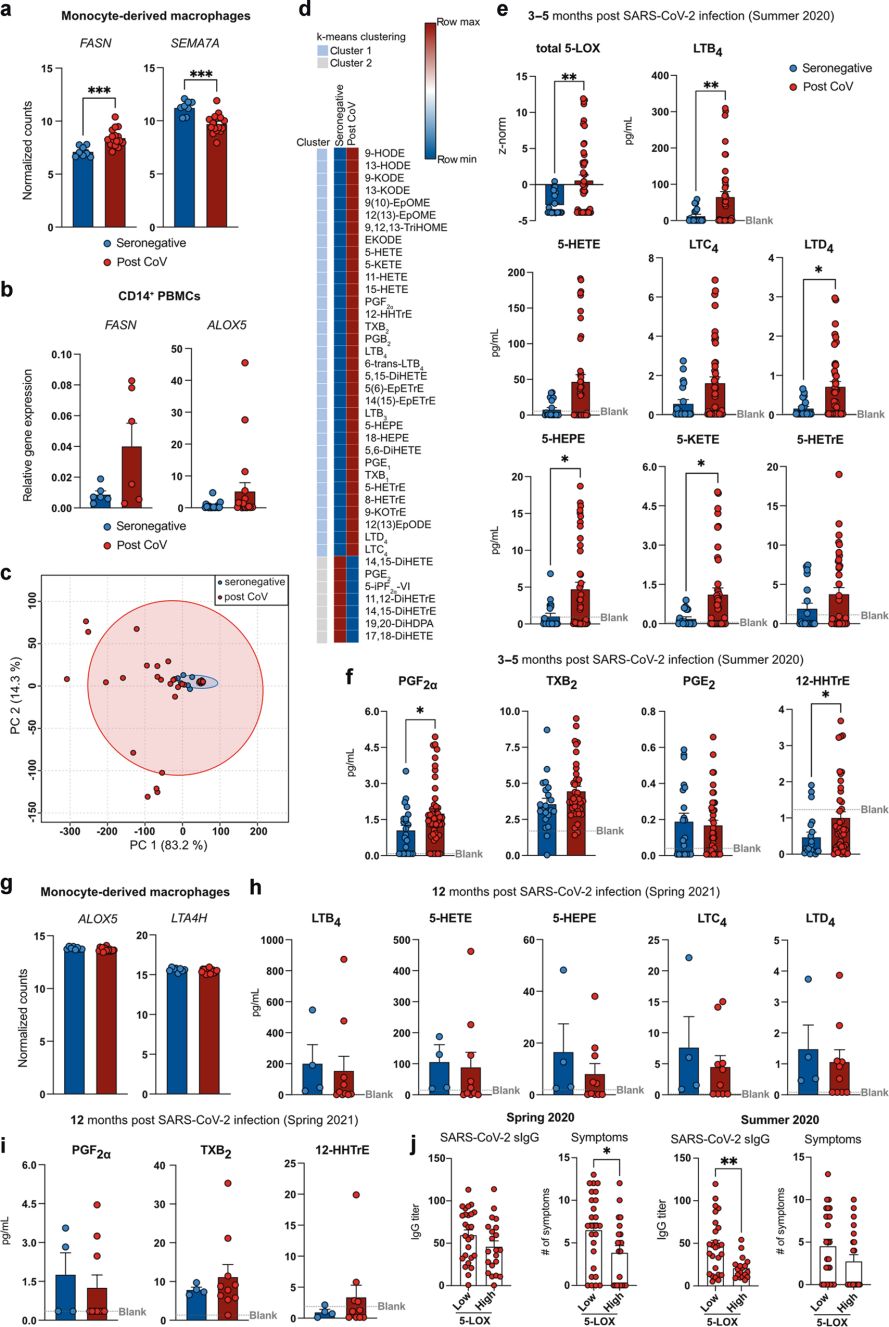
Post COVID-19 MDM produce increased amounts of inflammatory 5-lipoxygenase metabolites. **a** *FASN* and *SEMA7A* expression in seronegative (*n* = 8) and post CoV MDM (*n* = 16). **b** Gene expression of *FASN* and *ALOX5* in seronegative (*n* = 6/20) or post CoV (*n* = 6/19) CD14^+^ PBMCs. **c** PCA of lipid mediator profiles of MDM from seronegative (n = 22) or seropositive (n = 47) individuals. Red and blue circles: 95% CI. **d** Heatmap of lipid mediators produced by seronegative (*n* = 22) or post CoV (*n* = 47) MDM (LC-MS/MS). Clustering: with k-means using Pearson correlation. Data is shown as mean. **e** Sum of z-scored arachidonic acid derived 5-LOX metabolite concentrations for each donor. Levels of major 5-LOX (**e**, **h**) and COX (**f**, **i**) metabolites produced by MDM at 3-5 (**e**, **f**) or 12 (**h**, **i**) months p.i. (LC-MS/MS) shown as mean + SEM of *n* = 22/ *n* = 4 seronegative or *n* = 47/*n* = 10 seropositive individuals. **g** Expression of *ALOX5* and *LTA4H* (RNAseq) in MDM from seronegative (*n* = 8) or post CoV (*n* = 16) individuals. **j** IgG titers in serum or number of symptoms in MDM from post CoV donors stratified into 5-LOX low (z-score < 1) and high producers (z-score > 1). Bar graphs are depicted as mean + SEM. Statistical significance was determined by Mann–Whitney test. *p < 0.05; **p < 0.01; ***p < 0.001.

Previous studies had suggested an involvement of pro-inflammatory eicosanoids in severe, acute COVID-19^10,11,36^ and our RNAseq data indicated aberrant expression of genes involved in fatty acid and- eicosanoid synthesis in MDM and monocytes of convalescent, SARS-CoV-2 infected individuals (Figs. 1, 2, 3a, b, Table S1). Thus, we performed LC-MS/MS quantification of lipid mediators following stimulation with calcium ionophore to trigger PUFA mobilization and eicosanoid production. Compared to MDM from seronegative individuals, exhibiting considerable production of soluble epoxide hydrolase (sEH) metabolites (11,12-DiHETrE, 19,20-DiHDPA, 17,18-DiHETE), post COVID-19 MDM displayed broadly altered eicosanoid profiles that were dominated by pro-inflammatory 5-lipoxygenase (5-LOX) metabolites (Fig. 3c–e). Post COVID-19 MDM synthesized increased amounts of pro-inflammatory 5-LOX metabolites (LTB_4_, 5-KETE, 5-HEPE and LTD_4_), implicated in granulocyte chemotaxis and airway remodeling (Fig. 3d, e). In addition, the production of pro-inflammatory COX metabolites PGF_2α_ and 12-HHTrE was increased in post COVID-19 MDM (Fig. 3d, f).

This suggested that the prominent synthesis of inflammatory eicosanoids is not limited to acute and severe COVID-19^10,11^ and that reprogramming of innate immune cells may result in persistently enhanced LT production even following mild disease. Of note, we did not analyze spontaneous eicosanoid production, but used Ca^2+^ ionophore to elicit maximal eicosanoid responses, which allowed us to quantify lipid mediators in limited numbers of patient cells. Thus, eicosanoid profiles identified in the current study reflect a setting of acute inflammatory challenge. MDM of convalescent subjects also revealed a marked lower inferred soluble epoxide hydrolase activity. The epoxides of arachidonic acid have been reported to promote the resolution of inflammation, including mitigation of cytokine storms^37^. Accordingly, inhibition of the sEH has been proposed as a potential therapeutic target for COVID-19^38^. Our findings suggest that subsequent to mild COVID-19, MDM may exhibit a compensatory sEH activity that is shifted towards a pro-resolution state. In contrast to acute infection, which resulted in increased *ALOX5* expression in neutrophils and monocytes^10^, we did not find evidence of increased 5-LOX pathway gene expression in post COVID-19 MDM (Fig. 3g). Instead, genes involved in upstream events of fatty acid and lipid mediator biosynthesis (e.g., *FASN*, *DGAT2*, *PLA2G4C*) were upregulated in post COVID-19 MDM compared to MDM from seronegative subjects, suggesting an MDM phenotype in position for rapid activation of lipid metabolic pathways.

Analysis of MDM eicosanoid profiles from donors of the same cohort at 12 months post infection indicated that LT and prostanoid synthesis of post COVID-19 MDM had largely returned to baseline levels at this time point (Fig. 3h, i). This suggested that pro-inflammatory eicosanoid reprogramming in mild COVID-19 is transient, but that it may contribute to an enhanced inflammatory propensity during the first months post SARS-CoV-2 infection.

When stratified into 5-LOX low- or high producers, post COVID-19 subjects with high MDM LT production exhibited less acute symptoms but a faster decline in SARS-CoV-2 specific IgG titers (Fig. 3j), indicative of an efficient acute anti-viral response^39^. However, the lack of a defined clinical diagnosis of long COVID and poor reporting of long-term symptoms in the studied post COVID-19 cohort, prevented us from establishing a clear link between high MDM LT production and long-term symptoms of SARS-CoV-2 infection. Thus, future studies should investigate eicosanoid reprogramming in a cohort with clinically defined long COVID. Such studies would be imperative to define a potential pathological relevance of the inflammatory macrophage memory observed in the current study.

As patients in our study were enrolled following seroconversion, we were not able to compare monocyte and macrophage profiles at 3–5 months post infection to those during acute disease. However, we observed a considerable overlap between transcriptional profiles of post COVID-19 MDM and published transcriptomes of macrophages from SARS-CoV-2-infected individuals with mild acute disease^40^. Thus, several of the DEGs identified in our analysis (*MPEG1*, *CD163*, *CXCL9*, *MERTK*, and *MRC1*) were increased and correlated with higher expression of 5-LOX pathway genes in mild vs. severe acute disease^40^. It will be important to compare macrophage reprogramming between convalescent COVID-19 patients with different disease severities as well as following infection with other respiratory viruses (e.g., influenza). While previous studies have suggested an acute and transient increase in eicosanoids during respiratory syncytial virus (RSV) or influenza A virus (IAV) infection^41–43^, a comprehensive assessment of macrophage eicosanoid profiles in these diseases is currently lacking. PGE_2_ production was increased following IAV infection, however we did not observe increased PGE_2_ production in post COVID-19 MDM. Similarly, transcriptional profiles of post COVID-19 MDM showed minimal overlap with post influenza macrophage gene expression profiles^44,45^, suggesting that infection with different respiratory viruses results in distinct macrophage reprogramming. Increased macrophage LTB_4_ production may however contribute to protective immunity during acute infection with multiple respiratory viruses^41,43^. It will be important to determine, whether the persistent increase of LTB_4_ may contribute to a decreased susceptibility to respiratory viral infection during the first months following SARS-CoV-2 infection.

As airway inflammation, including in COVID-19, is commonly treated by glucocorticoids, we investigated potential effects of glucocorticoids on LT synthesis by post COVID-19 MDM. Fluticasone propionate, a commonly used inhaled glucocorticoid, further increased LT synthesis by post COVID-19 at baseline or after stimulation with house dust mite (HDM), used as a ubiquitous trigger of airway inflammation (Fig. S2a–d). This suggested that glucocorticoid treatment may further aggravate the pro-inflammatory eicosanoid reprogramming in post COVID-19 subjects. Given the therapeutic efficacy of glucocorticoids in airway inflammation, the finding that glucocorticoids enhanced LT synthesis may be surprising. However, it is in keeping with studies showing no reduction in LTs following glucocorticoid treatment in humans or enhanced LT production following in vitro treatment with glucocorticoids^46–48^.

### S-protein-triggered prostanoid response is enhanced in post COVID-19 MDM

**Fig. 4:**
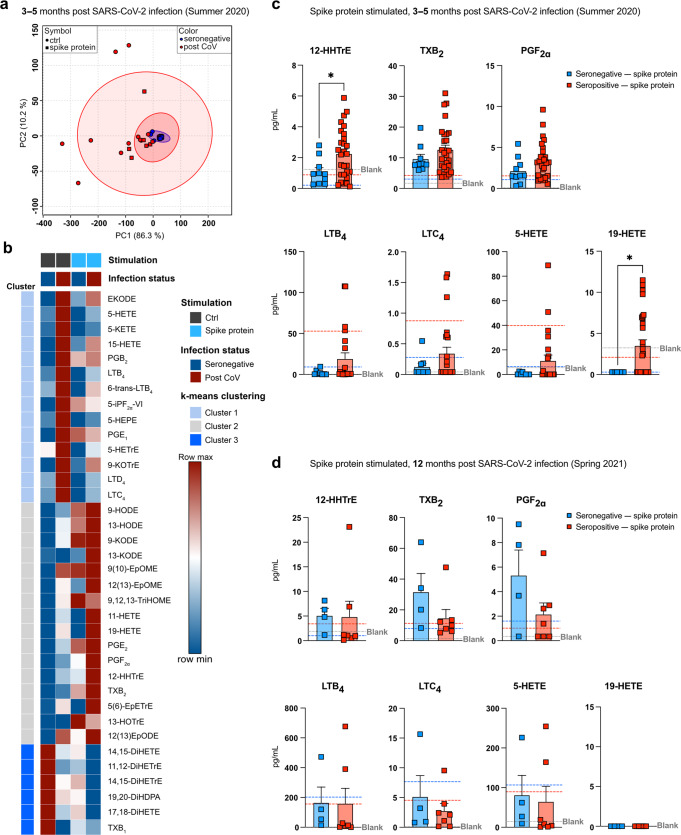
Increased S-protein-triggered prostanoid response in post COVID-19 MDM. **a** PCA of lipid mediators quantified in seronegative (*n* = 10) or post CoV (*n* = 29) MDM ± S-protein. Red and blue circles: 95% CI (LC-MS/MS at 3-5 months p.i). **b** Heatmap of lipid mediators produced by MDM (seronegative/ post CoV) ± S-protein; clustered with k-means using Pearson correlation. Data are shown as mean of seronegative (*n* = 10) or post CoV (*n* = 29) MDM. Concentrations of 12-HHTrE, TXB_2_, PGF_2α_ and 19-HETE/ 5-HETE produced by MDM + S-protein, at 3-5 months (**c**) or 12 months (**d**) p.i.; **d** *n* = 4 (seronegative); *n* = 7 (post CoV). Dashed lines indicate average ctrl level of either seronegative (blue) or seropositive (red) MDM. Bar graphs are depicted as mean + SEM. Statistical significance was determined by Mann–Whitney test. *p < 0.05.

To assess potential differences in eicosanoid production capacities under inflammatory conditions, we compared Ca^2+^ ionophore-elicited eicosanoid production in post COVID-19 and seronegative MDM stimulated for 24 h with S-protein or LPS. S-protein stimulation profoundly altered eicosanoid profiles (Fig. 4a, b), provoking a prominent induction of prostanoids from the thromboxane pathway (TXB_2_ and 12-HHTrE), while 5-LOX metabolites were reduced (Fig. 4b).

Compared to seronegative MDM, post COVID-19 MDM exhibited enhanced S-protein-induced prostanoid production, which was particularly evident for the thromboxane synthesis metabolite 12-HHTrE (Fig. 4b, c). Similarly, the cytochrome P450 metabolite 19-HETE was significantly increased in S-protein-stimulated post COVID-19, indicative of increased S-protein-mediated induction of vasoactive eicosanoids at 3-5 months post infection. In contrast at 12 months post infection, S-protein-triggered eicosanoid responses did not differ between SARS-CoV-2 seronegative and seropositive subjects (Fig. 4d). Compared to S-protein, LPS induced a stronger eicosanoid shift, thus overriding aberrant lipid mediator synthesis of post COVID-19 MDM (Fig. S3a, b). While upregulating prostanoids, LPS reduced the heighted production of LTD_4_ in post COVID-19 MDM (Fig. S3c), in line with suppressive effects of 24 h LPS stimulation on LT production by alveolar macrophages^49^. Together, this suggested that eicosanoid responses remain increased for several months following SARS-CoV-2 infection. In addition, during challenge with LPS or S-protein, eicosanoid profiles switch towards prostanoids with tissue reparative, vasoconstrictor and immune regulatory functions, potentially promoting repair of inflammation-induced tissue damage.

In contrast to eicosanoid profiles, cytokine production at baseline or following stimulation was not significantly different between post COVID-19 and seronegative MDM (Fig. S2e–h), suggesting that cytokine aberrations may not persist for >12 weeks or during monocyte-macrophage differentiation. However, in contrast to LTs, cytokine and prostanoid production by MDM was efficiently suppressed by fluticasone propionate (Fig. S2a–d, i). This suggested that cytokines and prostanoids are efficiently targeted, while exaggerated LT responses of post COVID-19 MDM are further exacerbated by glucocorticoids. Indeed, thromboxane is a major eicosanoid produced by inflammatory macrophages and involved in vascular and airway remodeling, thus its inhibition by glucocorticoids may provide a therapeutic benefit. However, glucocorticoids may in turn further enhance the heightened production of pro-inflammatory LTs by post COVID-19 MDM, thus promoting LT-driven airway inflammation and remodeling. Based on the enhanced production of 5-LOX-derived lipid mediators both in acute^10,11^ and post-acute COVID-19 (this study), approved LT pathway inhibitors should be considered as regimens to treat and/ or prevent airway inflammation and remodeling during the first 6 months following SARS-CoV-2 infection.

Future studies should further decipher upstream receptors and epigenetic pathways that drive the persistent pro-inflammatory macrophage and eicosanoid reprogramming during SARS-CoV-2 infection. In addition, a potential heterogeneity in GM-CSF and TGF-β1-differentiated MDM from seronegative and seropositive individuals should be addressed in single cell analyses. LTs have been reported to induce CCL2 in monocytes^50,51^, suggesting that enhanced LT synthesis may drive exaggerated pro-inflammatory chemokine responses in post COVID-19 MDM. In turn, increased CCL2 production by post COVID-19 MDM or monocytes may promote LTB_4_ production^52^. Thus, our combined RNAseq and LC-MS/MS data suggest a crosstalk between CCL2 and LTs, which perpetuates the persistent pro-inflammatory activation of monocytes and macrophages following SARS-CoV-2 infection. Due to limitations in patient material, we could not perform a comprehensive comparison of MDM and monocytes, however our data suggest that differences in CCL2 and fatty acid synthesis are at least partially present in undifferentiated post COVID-19 monocytes, which differentiate into inflammatory monocyte-derived macrophages when entering the lung^5^. The persistent upregulation of pro-inflammatory eicosanoids in post COVID-19 macrophages may have multiple consequences for subsequent immune responses, e.g. during bacterial or viral infection or in patients suffering from chronic inflammatory diseases such as asthma, thus requiring future investigation.

## Methods

### Study design

Symptoms of seronegative (SARS-CoV-2 seronegative) and post COVID-19 (SARS-CoV-2 seropositive) individuals were determined through a questionnaire in Spring 2020 and 3–5 months later, in Summer 2020. Percentage of each symptom was calculated separately for seropositive and -negative individuals (table S1). Sample sizes for each experiment are specified in the corresponding figure legends; an overview is depicted in Fig. S1a. All blood donors participated in the study after informed written consent. All procedures were approved by the local ethics committee at the University clinic of the Technical University of Munich (internal references: 216/20S, 263/21S) and in accordance with the declaration of Helsinki.

### Monocyte-derived macrophage culture

Isolated peripheral blood mononuclear cells (PBMCs) of post COVID-19 or seronegative individuals were used to generate monocyte-derived macrophages (MDM), as previously reported^53,54^. MDM were cultured in the presence of 10 ng/mL human GM-CSF (Miltenyi Biotec, Bergisch-Gladbach, Germany) and 2 ng/mL human TGF-β (Peprotech, Hamburg, Germany). After 7 days incubation, cells were harvested and stimulated for 24 h with 100 ng/mL LPS (Invivogen, San Diego, CA, USA), 20 nM spike protein (antibodies-online GmbH), 10 µg/mL house dust mite extract (HDM) (Citeq Biologics, Groningen, The Netherlands), 1 µM fluticasone propionate (FP) (Sigma-Aldrich, St. Louis, MO, USA), 5 µM or 100 nM dexamethasone (DXM) (Sigma-Aldrich, Merck). After 24 h of stimulation cells were harvested in presence of Ca^2+^-ionophore A23187 (Sigma-Aldrich, Merck).

### NHBE and ALI culture

Primary normal human bronchial epithelial cells (NHBEs) (Lonza, Basel, Switzerland) from non-smokers in passage 3 were grown to 80–90% confluency in Bronchial Epithelial Cell Growth Medium (BEGM) (Lonza). Following starvation overnight in bronchial epithelial basal medium (BEBM) (Lonza), NHBEs were stimulated for 24 h with 1 µg/mL HDM (Citeq) or 1 µM FP (Sigma-Aldrich, Merck). For air-liquid interface (ALI) cultures, NHBEs were split at 60–80% confluency and 1 × 10^5^ cells were seeded on 12 mm transwells (0.4 μm pores, Stemcell Technologies, Vancouver, Canada). Cultures were maintained in BEGM (500 µL apical and 1000 µL basal) until cells reached full confluency. Subsequently, cells were “airlifted” by removing the apical medium, and basal medium was replaced with PneumaCult-ALI Maintenance Medium (Stemcell Technologies). Medium was replaced every 2 days and excessive mucus washed away with DPBS (Gibco). Cells were cultured at air liquid interface for 3–4 weeks. Before stimulation, cells were starved overnight in PneumaCult-ALI Basal Medium (Stemcell Technologies). ALI cells were stimulated on the apical side with 1 µg/mL HDM (Citeq), 1 µM FP (Sigma-Aldrich) or corresponding control for 24 h.

### Histology

For histology ALI cells were fixed in 4% formaldehyde and embedded in paraffin. Sections were cut and hematoxylin & eosin (H&E) stained at the Klinikum rechts der Isar, Dermatology Department.

### RNA isolation

Cells were lysed in RLT buffer (Qiagen, Hilden, Germany) supplemented with 1% β-mercaptoethanol. RNA was extracted using a spin-column kit according to the manufacturer’s instructions (Zymo Research, Freiburg, Germany) and transcribed into DNA using the HighCapacity cDNA Reverse Transcription kit according to the manufacturer’s instructions (Applied Biosystems) or submitted for total RNA sequencing.

### RNA sequencing

Library preparation was performed using the TruSeq Stranded mRNA Library Prep Kit (Illumina, San Diego, CA, USA). Briefly, RNA was isolated from MDM cell lysates according to the manufacturer’s instructions (Zymo Research). Total RNA quality and quantity was assessed by Qubit 4 Fluorometer (Invitrogen) and RNA integrity number (RIN) was determined with the Agilent 2100 BioAnalyzer (RNA 6000 Nano Kit, Agilent).

For library preparation, 1 μg of RNA was poly(A) selected, fragmented, and reverse transcribed with the Elute, Prime, Fragment Mix (Illumina). A-tailing, adaptor ligation, and library enrichment were performed as described in the TruSeq Stranded mRNA Sample Prep Guide (Illumina). RNA libraries were assessed for quality and quantity with the Agilent 2100 BioAnalyzer and the Quant-iT PicoGreen dsDNA Assay Kit (Life Technologies, Thermo Fisher Scientific). RNA libraries were sequenced as 150 bp paired-end runs on an Illumina NovaSeq 6000 platform. Sequencing was performed at the Helmholtz Zentrum München (HMGU) by the Genomics Core Facility.

### Cytokine analysis (ELISA)

Cell culture supernatants were analyzed for IL-6, IL-1β and IL-8 secretion using the human ELISA Sets (BD Biosciences, Franklin Lakes, NJ, USA) according to the manufacturer’s instructions.

### Lipid mediator quantification

Briefly, cell supernatants from 200,000 cells, stored in equal volume of methanol, were extracted using solid phase extraction (Evolute Express ABN, Biotage, Uppsala, Sweden) and lipid mediators (see Table S1) were quantified by liquid chromatography coupled to tandem mass spectrometry (LC-MS/MS)^55^. Given that cell culture media has significant background levels of many lipid mediators, compounds whose concentration was below the media level were excluded from data analysis.

### Real-time quantitative PCR

10 ng cDNA was used as a template. The list of applied primers (4 µmol/L, Metabion international AG, Planegg, Germany) can be found in the Supplement. FastStart Universal SYBR Green Master Mix (Roche, Basel, Switzerland) was used and fluorescence was measured on a ViiA7^TM^ Real-Time PCR System (Applied Biosystems, Thermo Fisher Scientific). The expression levels were normalized to the house-keeping genes GAPDH (for MDM), ACTB, HPRT1 and TFRC *(average for NHBEs and ALI cultured cells)*. Relative gene expression was calculated as 2^ΔCT^ (ΔC_T_ = C_T (Housekeeper)_ - C_T(Gene)_). For genes where expression could not be quantified, CT values were set to 40.

### Data analysis and statistics

LC-MS/MS and RNAseq data were analyzed using previously published procedures^14,48,55,56^. Details can be found in the Supplement.

## Supplementary information

Table S1

Supplementary information 1
