# Supplementary material for: Mild COVID-19 imprints a long-term inflammatory eicosanoid- and chemokine memory in monocyte-derived macrophages
Source: Mucosal Immunol. 2022 Mar 15;15(3):515–24. doi: 10.1038/s41385-021-00482-8 (PMC9038526; doi:10.1038/s41385-021-00482-8)
Supplement: Supplementary file 2 — Supplementary information 1 [file 41385_2021_482_MOESM2_ESM.pdf]

Supplemental Information, Bohnacker et al.

Supplementary Methods

Primer sequences used for qPCR:

Human Primer	Forward Sequence	Reverse Sequence
ACE2	GGGATCAGAGATCGGAAGAAGAAA	AGGAGGTCTGAACATCATCAGTG
ACOD1	TGCCAAAGAGATTCCATCCCC	CTCGGCACTTTGTCGAGCTA
ACTB	GGATGCAGAAGGAGATCACT	CGATCCACACGGAGTACTTG
ALOX5	GATTGTCCCCATTGCCATCC	AGAAGGTGGGTGATGGTCTG
CMPK2	CACCCGGTGGTGCCAG	GAGGTACACTCCTCCAAAACG
CXCL8	GAAGTTTTTGAAGAGGGCTGAGA	TGCTTGAAGTTTCACTGGCAT
EIF2AK2	GCATGGGCCAGAAGGATTTC	GCGGCCAATTGTTTTGCTTC
GAPDH	GAAGGTGAAGGTCGGAGT	GAAGATGGTGATGGGATTTC
HPRT1		
IDO1	ACAGACCACAAGTCACAGCG	TTGGCAAGACCTTACGGACA
IFIH1	AGATGCAACCAGAGAAGATCCA	TGGCCCATTGTTCATAGGGT
IL1B	AGAAGTACCTGAGCTCGCCA	CTGGAAGGAGCACTTCATCTGT
IL6	ACATGTGTGAAAGCAGCAAAG	GGCAAGTCTCCTCATTGAATCC
IRF7	CATCTTCAAGGCCTGGGCTG	GCAGCGGAAGTTGGTTTTCC
ISG15	CAGCGAACTCATCTTTGCCAG	GGACACCTGGAATTCGTTGC
ISG20	GTGGCCAGGCTAGAGATCC	GGAAGTCGTGCTTCAGGTCA
LTC4S	GACGGTACCATGAAGGACGA	GGAGAAGTAGGCTTGCAGCAG
OAS2	TCAACGCTCTGAGCTTAAATGA	CAAACTCACCAGGACTGGCA
OAS3	ACTACAACGCCAAGGACAAGA	GATGATAGGCCTGGGCTTCTG
OASL	GGACATCGTTGCTCAGAGGG	CTCGTGCCCTCTTCACGTT
PTGDS	CCCAGGGCTGAGTTAAAGGA	AGAGCAGAGACATCCAGAGC
PTGES	TCAAGATGTACGTGGTGGCC	GAAAGGAGTAGACGAAGCCCAG
PTGS2	GCTGGAACATGGAATTACCCA	CTTTCTGTACTGCGGGTGGAA
TFRC	GGACGCGCTAGTGTTCTTCT	CATCTACTTGCCGAGCCAGG
TMPRSS2	AGAGTGCGACTCCTCAGGTA	AGGCGAACACACCGATTCTC
USP18	GGCTCCTGAGGCAAATCTGT	CAACCAGGCCATGAGGGTAG

HPRT1: QuantiTect Primer Assay Cat.No. QT00059066 (Qiagen)

Flow cytometry

Human MDM from either seronegative or seropositive patients were stained with antibodies against EP2 (PE), HLA-DR (BV650), CD206 (BB515) (all from BD Biosciences) as well as CD11b (BV421) (Biolegend). All samples were acquired on a BD LSRFortessa (BD Biosciences) and analyzed by using FlowJo v10 software (FlowJo LLC).

Data analysis and statistics

For LC-MS/MS (lipid mediator) and ELISA (cytokines/ chemokines) data, missing values below the lower limit of detection were interpolated using ¼ of the minimum value for each metabolite. For the lipid mediator dataset outliers were excluded using the ROUT (robust regression and outlier removal) method for each stimulation separately in Graph Pad Prism (version 9). Metabolites with more than 60% of values below the media blank levels were excluded. Statistical analysis of two group comparisons was performed either using Mann-Whitney (unpaired) or Wilcoxon test (paired). For multiple comparisons, Kruskal-Wallis test with Dunn correction was chosen. Principal component analyses (PCA) were generated using MetabolAnalyst 5.0.[1] The Morpheus software (Broad Institute) was used for the design of heatmaps.

RNAseq data were analyzed in R. Genes with fewer than 100 counts in the RNA-seq data set were filtered out. Only the genes with more than 10 counts in at least one sample were kept. Next, the vst[2] normalization implemented in the DESeq2 package[3] was used to normalize the filtered RNA-seq counts. Combat[4] was used for batch correction implemented in the sva package[5] in order to remove the batch effects of gender and age. During the batch correction, age was categorized into groups of 5 years intervals. Principal component analysis (PCA) on the normalized and batch-corrected data was performed and the ggplot2 package[6] was used to plot the samples in the plane on the 1st and 2nd principal components. The DESeq2 package was used to calculate the fold change, base mean and adjusted p-values of the differentially genes for the following comparisons: seronegative without any treatment vs post CoV without any treatment; seronegative treated with spike vs post CoV treated with spike; and seronegative treated with LPS vs post CoV treated with LPS. The pheatmap package[7] (https://CRAN. R-project. org/package= pheatmap) was used to plot heatmaps of the top 50 genes with the largest log2 fold change with at least 50 base counts for the above-mentioned comparisons. The genes in rows of the heatmaps were not clustered, while the samples in the columns of the heatmaps were clustered based Euclidean distance. The EnhancedVolcano package[8] (https://github.com/kevinblighe/EnhancedVolcano) was used to create volcano plots of the differentially expressed genes with at least 50 base counts.

Supplementary Figures

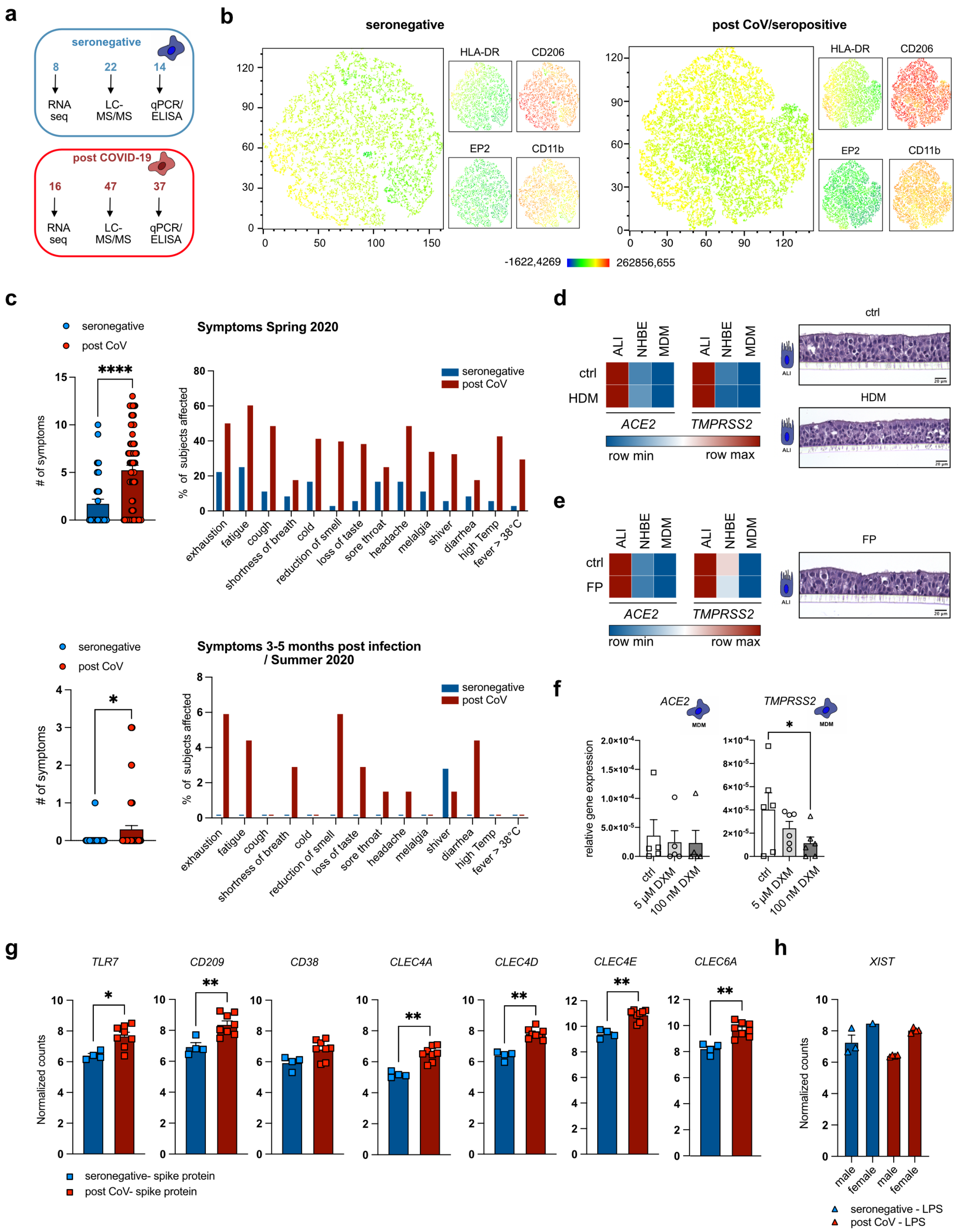

Figure S1: Monocyte-derived macrophages express low levels of ACE2 and TMPRSS2, but high levels of C-type lectin receptors involved in SARS-CoV-2 recognition

(a) Overview of samples from the post COVID cohort and the corresponding seronegative patients that were subjected to each type of analysis. (b) Representative FACS data (tsne plots) for MDM from seronegative (n=2) or post CoV/seropositive subjects (n=4). (c) Symptom load of seronegative (healthy) (n=36) and SARS-CoV-2 seropositive (post CoV) (n=68) individuals in Spring 2020 or at 3-5 months post infection (Summer 2020). (d) Relative gene

expression (shown as average in the heatmap) of *ACE2* and *TMPRSS2* at baseline and upon house dust mite extract (HDM) stimulation (10 µg/mL) in ALI (n=5), NHBE (n=3) and MDM (n=4) measured by qPCR. Hematoxylin & eosin staining of ALI cells after stimulation with HDM (10 µg/mL). (e) Relative gene expression (shown as average in the heatmap) of *ACE2* and *TMPRSS2* in ALI (n=5), NHBE (n=3) and MDM (n=4) after fluticasone propionate (FP) treatment (1 µM). (f) Effect of dexamethasone (DXM) on *ACE2* and *TMPRSS2* expression in healthy MDM. Healthy volunteers n=4 included in panels d-f were recruited in a cohort separate from the post COVID cohort. (g) Expression of pathogen recognition receptors (RNAseq) upon spike protein stimulation in MDM from seronegative (n=4) or post COVID (post CoV, n=8) individuals. Bar graphs are depicted as mean + SEM. Statistical significance was determined by Mann-Whitney test (g) or Kruskal-Wallis test followed by Dunn's multiple comparison test (f,h). *P < 0.05; **P < 0.01; ***P < 0.001; ****P < 0.0001.

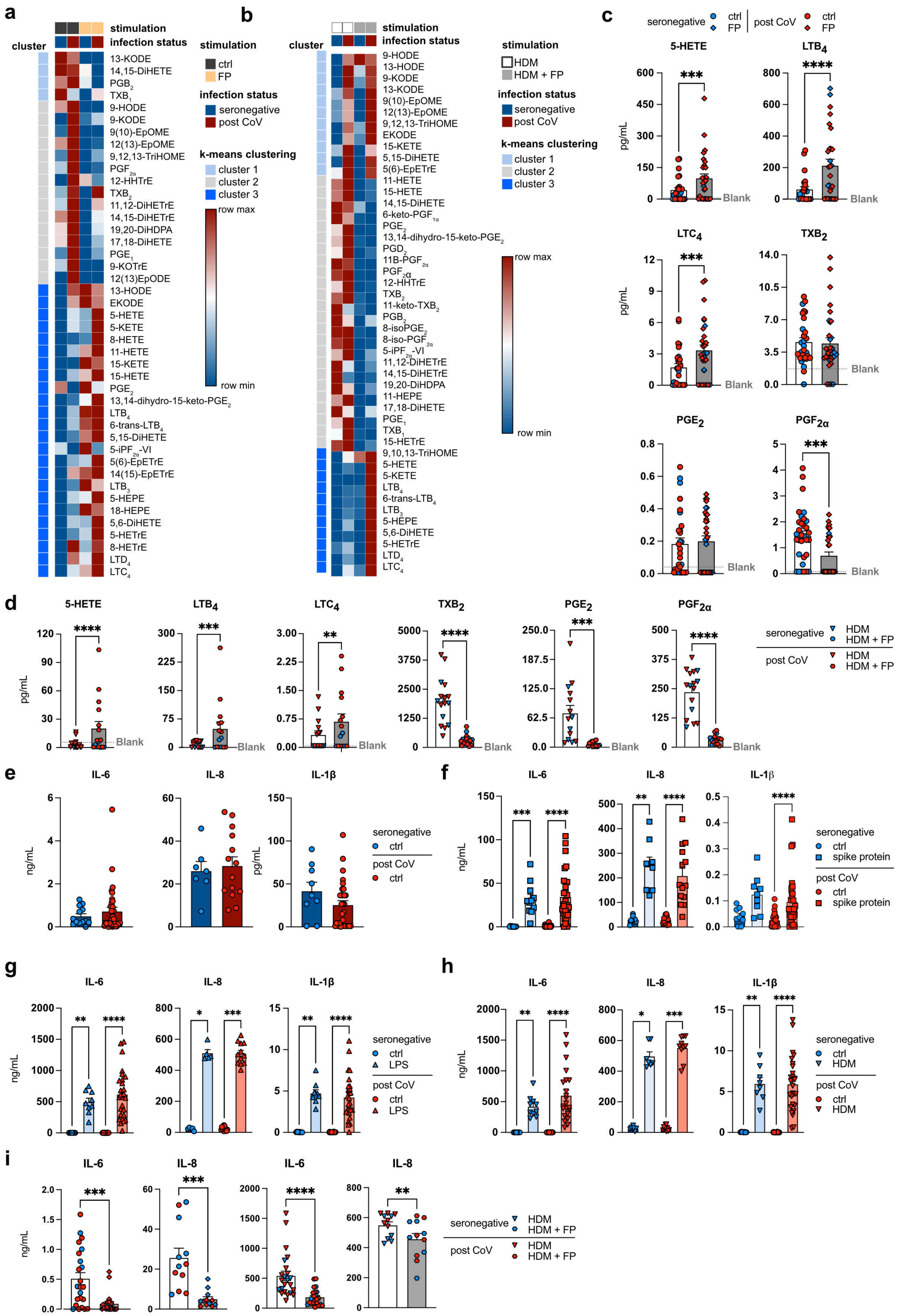

a
stimulation
infection status
cluster
13-KODE
14,15-DiHETE
PGB2
TXB1
9-HODE
9-KODE
9(10)-EpOME
12(13)-EpOME
9,12,13-TriHOME
PGF2a
12-HHTrE
TXB2
11,12-DiHETrE
14,15-DiHETrE
19,20-DiHDPA
17,18-DiHETE
PGE1
9-KOTrE
12(13)EpODE
13-HODE
EKODE
5-HETE
5-KETE
8-HETE
11-HETE
15-KETE
15-HETE
PGE2
13,14-dihydro-15-keto-PGE2
LTB4
6-trans-LTB4
5,15-DiHETE
5-iPF2a-VI
5(6)-EpETrE
14(15)-EpETrE
LTB3
5-HEPE
18-HEPE
5,6-DiHETE
5-HETrE
8-HETrE
LTD4
LTC4
stimulation
ctrl
FP
infection status
seronegative
post CoV
k-means clustering
cluster 1
cluster 2
cluster 3
row max
row min
b
stimulation
infection status
cluster
9-HODE
13-HODE
9-KODE
13-KODE
9(10)-EpOME
12(13)-EpOME
9,12,13-TriHOME
EKODE
15-KETE
5,15-DiHETE
5(6)-EpETrE
11-HETE
15-HETE
14,15-DiHETE
6-keto-PGF1a
PGE2
13,14-dihydro-15-keto-PGE2
PGD2
11B-PGF2a
PGF2α
12-HHTrE
TXB2
11-keto-TXB2
PGB2
8-isoPGE2
8-iso-PGF2a
5-iPF2a-VI
11,12-DiHETrE
14,15-DiHETrE
19,20-DiHDPA
11-HEPE
17,18-DiHETE
PGE1
TXB1
15-HETrE
9,10,13-TriHOME
5-HETE
5-KETE
LTB4
6-trans-LTB4
LTB3
5-HEPE
5,6-DiHETE
5-HETrE
LTD4
LTC4
stimulation
HDM
HDM + FP
infection status
seronegative
post CoV
k-means clustering
cluster 1
cluster 2
cluster 3
row max
row min
c
seronegative ctrl FP
post CoV ctrl FP
5-HETE
LTB4
LTC4
TXB2
PGE2
PGF2α
pg/mL
Blank
d
5-HETE
LTB4
LTC4
TXB2
PGE2
PGF2α
pg/mL
Blank
seronegative HDM HDM + FP
post CoV HDM HDM + FP
e
IL-6
IL-8
IL-1β
ng/mL
pg/mL
seronegative ctrl
post CoV ctrl
f
IL-6
IL-8
IL-1β
ng/mL
seronegative ctrl spike protein
post CoV ctrl spike protein
g
IL-6
IL-8
IL-1β
ng/mL
seronegative ctrl LPS
post CoV ctrl LPS
h
IL-6
IL-8
IL-1β
ng/mL
seronegative ctrl HDM
post CoV ctrl HDM
i
IL-6
IL-8
IL-6
IL-8
ng/mL
seronegative HDM HDM + FP
post CoV HDM HDM + FP

Figure S2: Corticosteroids differentially affect prostanoid, leukotriene and cytokine production in post COVID-19 macrophages

(a,b) Heatmap of lipid mediators quantified by LC-MS/MS in supernatant of MDM stimulated +/- fluticasone propionate (FP) (1 µM), house dust mite extract (HDM) (10 µg/mL) and HDM+FP. Rows are clustered with k-means using Pearson correlation. Data are shown as mean of seronegative (n=5) or SARS-CoV-2 seropositive (n=11) individuals. (c-d) 5-LOX and COX metabolites produced by MDM (seronegative or post CoV) stimulated with +/- fluticasone propionate FP (1 µM), HDM (10 µg/mL) or HDM+FP. (e-i) IL-6, IL-8 and IL-1β secretion in supernatant of MDM from seronegative or SARS-CoV-2 seropositive (post CoV) individuals; (e) Baseline (ctrl), (f) after stimulation with S-protein (20 nM), (g) LPS (100 ng/mL), (h) house dust mite extract (HDM) (10 µg/mL), (i) FP (1 µM) or HDM+FP quantified by ELISA. Data are shown as mean + SEM. Seronegative individuals (n=8-14), previously SARS-CoV-2 infected individuals (n=13-37). Statistical significance was determined by Mann-Whitney test (c-e; i) or Kruskal-Wallis test followed by Dunn’s multiple comparison test (f-h). *P < 0.05; **P < 0.01; ***P < 0.001; ****P < 0.0001.

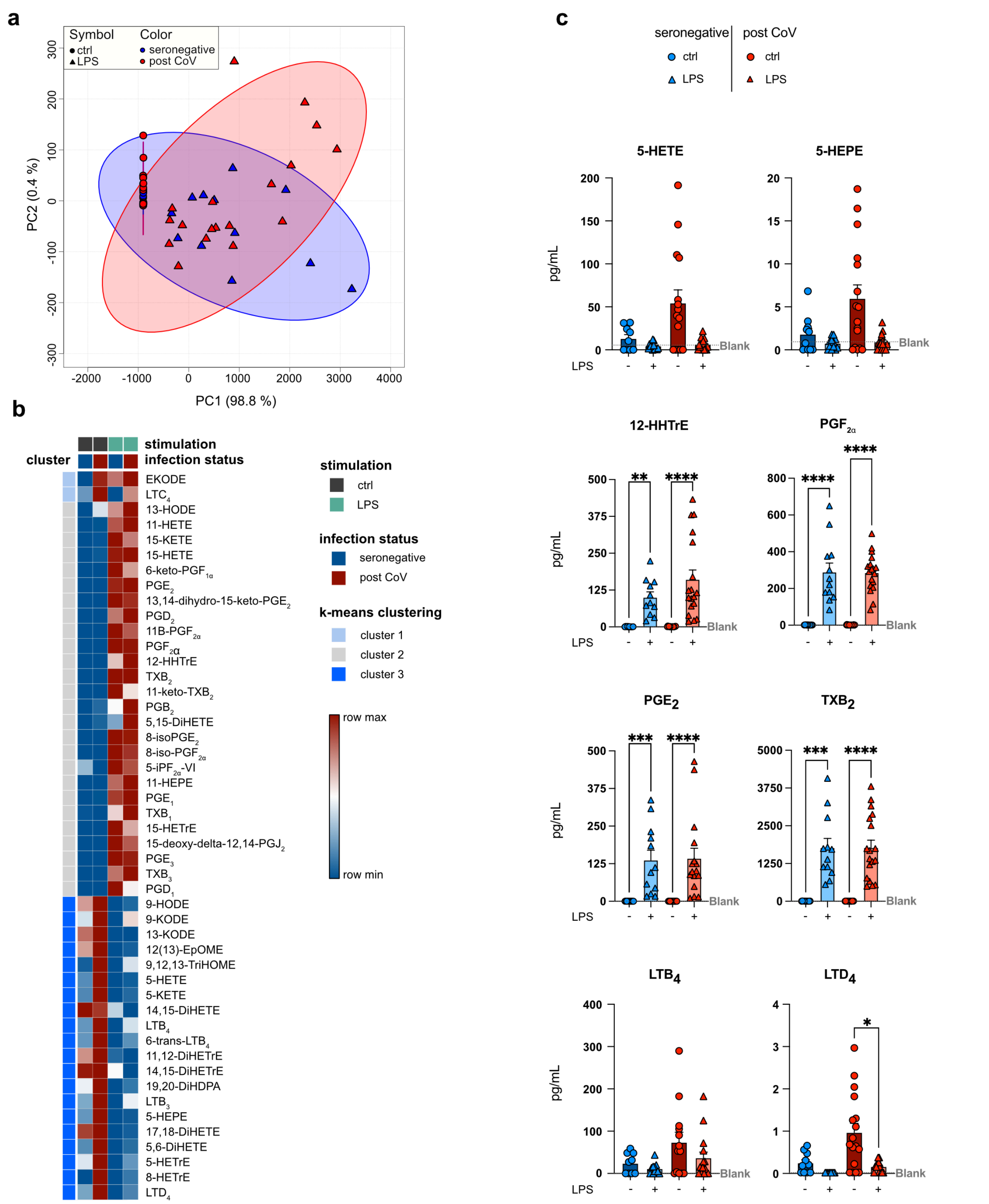

Figure S3: LPS-induced eicosanoid responses are largely unaltered in post COVID-19 MDM

(a) PCA of lipid mediators quantified in MDM supernatant (SN) of seronegative (n=12) or SARS-CoV-2 seropositive (n=18) individuals +/- LPS (100 ng/mL) stimulation. Red and blue circles depict the 95% CI. (b) Heatmap of lipid mediators measured in MDM SN stimulated +/- LPS. Rows are clustered with k-means using Pearson correlation. Data are shown as mean for MDM from seronegative (n=12) or SARS-CoV-2 seropositive (n=18) individuals. (c) Concentrations of major COX and 5-LOX metabolites in MDM SN after 24h stimulation with LPS quantified by LC-MS/MS, depicted as mean + SEM of n=12 (seronegative) or n=18 (post

CoV). Statistical significance was determined by Kruskal-Wallis test followed by Dunn's multiple comparison test. *P < 0.05; **P < 0.01; ***P < 0.001; ****P < 0.0001.

Supplementary Tables:

Table S1: Patient characterization and differentially expressed genes in MDM from seropositive and seronegative individuals +/- stimulation with LPS or S-protein; subjects characteristics for each figure separately; metabolites included in the LC-MS/MS panel

References

1. Chong, J., Wishart, D. S. & Xia, J. Using MetaboAnalyst 4.0 for Comprehensive and Integrative Metabolomics Data Analysis. *Current Protocols in Bioinformatics* **68**, (2019).
2. Anders, S. & Huber, W. Differential expression analysis for sequence count data. *Genome Biol* **11**, R106 (2010).
3. Love, M. I., Huber, W. & Anders, S. Moderated estimation of fold change and dispersion for RNA-seq data with DESeq2. *Genome Biol* **15**, 550 (2014).
4. Johnson, W. E., Li, C. & Rabinovic, A. Adjusting batch effects in microarray expression data using empirical Bayes methods. *Biostatistics* **8**, 118–127 (2007).
5. Leek, J. T., Johnson, W. E., Parker, H. S., Jaffe, A. E. & Storey, J. D. The sva package for removing batch effects and other unwanted variation in high-throughput experiments. *Bioinformatics* **28**, 882–883 (2012).
6. Wickham *ggplot2: Elegant Graphics for Data Analysis*. (Springer-Verlag New York, 2016).
7. Kolde R. pheatmap: Pretty heatmaps [Software]. (2015).at <https://CRAN. R-project. org/package= pheatmap>
8. Blighe K *EnhancedVolcano: Publication-ready volcano plots with enhanced colouring and labeling*. (2019).at <https://github.com/kevinblighe/EnhancedVolcano>
